# TRACE: reconstructing fragmented microbial landscapes for high-resolution antimicrobial resistance surveillance

**DOI:** 10.3389/fmicb.2026.1795951

**Published:** 2026-03-17

**Authors:** Xiaolu Bai, Jinli Zhang, Zongli Jiang, Zhihan Jiang, Haoran Liu

**Affiliations:** College of Computer Science, Beijing University of Technology, Beijing, China

**Keywords:** antimicrobial resistance, clinical narratives, digital phenotypes, infectious disease characterization, systems microbiology

## Abstract

**Introduction:**

Comprehensive mapping of Antimicrobial Resistance (AMR) dynamics is a cornerstone of systems microbiology and global health security. However, the precise characterization of microbial interactions within clinical ecosystems is severely confined by the fragmentation of phenotypic evidence. Critical data points, including pathogen identity, anatomical niche, and susceptibility profiles, are often buried within unstructured clinical narratives. This fragmentation creates a blind spot in surveillance, where the ecological signals of rare but critical resistance phenotypes are lost, preventing a systems-level understanding of microbial evolution.

**Methods:**

To bridge this gap, we propose the Triple-based Reconstruction for Antimicrobial Clinical Evidence (TRACE) framework, a systems-computational approach that treats clinical records as unstructured microbiological sensors. Unlike traditional classification models, our approach focuses on the biological reconstruction of evidence. It first aggregates scattered findings into coherent Organism-Specimen-Susceptibility biological clusters. To resolve the scarcity of data for underrepresented pathogens, which represent the long-tail of biodiversity, we employ a constraint-based *in silico* simulation strategy. This component utilizes a large language model to synthesize biologically plausible phenotypic profiles that are rigorously verified for factual consistency. Finally, the framework employs parameter-efficient adaptation to capture the subtle semantic nuances of complex resistance patterns.

**Results:**

Validated on the MIMIC-III and MIMIC-IV datasets, TRACE demonstrates a superior ability to recover lost microbial signals. On the challenging MIMIC-IV benchmark, it achieves a Macro-F1 score of 15.4% and a Precision@5 of 70.3%. More importantly, the system successfully disentangles clinically similar but biologically distinct resistance phenotypes, significantly reducing the misidentification of rare pathogen-drug combinations.

**Conclusion:**

By resolving evidence fragmentation and effectively expanding the feature space for underrepresented microorganisms, TRACE provides a robust solution for fine-grained infectious disease characterization. This framework establishes a foundation for high-precision AMR phenotype identification, paving the way for more reliable monitoring of resistance trends in clinical ecosystems.

## 1 Introduction

The systemic analysis of pathogenic microorganisms within clinical environments is pivotal for understanding the temporal and spatial dynamics of infectious diseases. The clinical ecosystem functions as a complex reservoir where microbial evolution accelerates under selective pressure. Within this context, the precise characterization of microbiological parameters, particularly the Organism-Specimen-Susceptibility (O-S-S) triad, is indispensable. It serves not only therapeutic management but also the broader objectives of systems microbiology, which include mapping the trajectory of hospital-acquired infections, conducting global antimicrobial resistance (AMR) surveillance, and tracking pathogen adaptation pathways (Jiang et al., 2022). Consequently, International Classification of Diseases (ICD) codes, when viewed as standardized digital phenotypes, offer a unique opportunity to map these biological states at a population scale (Kumar et al., 2024). However, the utility of these digital phenotypes for systems-level analysis is currently compromised by the fidelity with which microbiological evidence is captured from the noise of Electronic Health Records (EHR).

In real-world clinical corpora, microbe-rela ted data exhibit a pronounced long-tail distribution, reflecting the natural biodiversity of pathogens. While common pathogens dominate the dataset, a substantial number of fine-grained combinations involving specific microorganisms and rare resistance profiles (herein referred to as O-S-S) are sparsely represented (Kakizaki et al., 2023). This sparsity poses a fundamental challenge to data-driven modeling. Existing literature on automated ICD coding has documented both the extensive scale of the label space and the prevailing tendency to evaluate systems predominantly on frequent codes, thereby obscuring significant performance deficits regarding clinically critical rare labels (Teng et al., 2023). Furthermore, while mitigation strategies such as re-weighted loss functions (Ridnik et al., 2021) or hierarchical label exploitation (Wang et al., 2024) have been proposed, they address class imbalance primarily from a statistical perspective; they cannot generate missing evidence. If the signal for a rare pathogen is absent or unrecognizable in the source text, loss re-weighting alone cannot enable a model to correctly predict the corresponding code. While low disease prevalence is a contributing factor, our analysis reveals a more fundamental, often overlooked determinant: a critical “supply shortage” of explicit microbiological evidence within clinical narratives. Specifically, the evidence required to map these fine-grained codes is frequently fragmented, heterogeneous, or obscured by noise, leading to three distinct failure mechanisms:

First, critical evidence is characterized by high sparsity and susceptibility to signal dilution. In a typical discharge summary, sentences containing definitive microbiological facts (e.g., “Urine culture positive for E. coli”) constitute a negligible fraction of the total text. Full-text models are frequently overwhelmed by non-pathogenic “noise”, such as extensive medical history or unrelated prescriptions. Consequently, models fail to extract stable patterns from this noisy context, often defaulting to broader “parent codes” based on co-occurring clinical features while overlooking the specific pathogen evidence essential for granular coding.

Second, the combinatorial complexity of the O-S-S triad exacerbates data fragmentation. The multiplicative combinations of distinct pathogens, specimen types, and susceptibility profiles result in a label space where valid combinations far exceed available training samples. While general few-shot learning frameworks (Zhang et al., 2023) attempt to facilitate knowledge transfer, they struggle to capture the specific semantics of these dispersed O-S-S triplets. The lack of aggregated evidence leads to high variance in parameter estimation, causing models to suffer from “degenerative prediction,” wherein specific rare resistance profiles are misclassified as common sensitive strains–a critical error for AMR monitoring.

Third, textual heterogeneity challenges conventional augmentation methods. Uncontrolled generation from unstructured clinical text risks introducing fabrications, such as spurious pathogen identities or susceptibility profiles, which are intolerable in clinical surveillance (Falis et al., 2024; Wu et al., 2025). Therefore, robust frameworks capable of structuring and enriching fragmented microbial data are essential for the advancement of computational systems microbiology.

Prior efforts have addressed specific facets of these challenges, yet significant gaps remain. Early rule-based and classical machine-learning approaches relied on hand-crafted rules or TF-IDF features combined with SVMs; however, these methods failed to scale effectively to the extensive ICD label space (Zhang et al., 2025a; Perotte et al., 2014). Deep learning methodologies subsequently introduced segment-level attention and hierarchical encoders to capture local clinical cues, but continued to struggle with extreme label sparsity and the voluminous, unstructured nature of clinical narratives (Zhang et al., 2025b; Liu et al., 2021; Michalopoulos et al., 2022). Later studies explored long-document architectures and domain-specific pretraining, such as BioBERT and ClinicalBERT, to enhance biomedical language representation. Concurrent research examined loss re-weighting, hierarchical label structures, and label-correlation modeling as strategies to mitigate class imbalance (Huang et al., 2022; Chomutare et al., 2022; Zhang et al., 2021).

At the data level, researchers have proposed lexical augmentation, ontology-guided substitution, and, more recently, Large Language Model (LLM)-based synthetic note generation to expand rare classes. However, uncontrolled synthesis may introduce factual inconsistencies or medical hallucinations, posing a particularly problematic issue for microbiology-sensitive ICD labels that demand precise organism, specimen, and susceptibility data (Feng, 2025; Falis et al., 2022; Wu et al., 2025). In parallel, parameter-efficient adaptation methods, such as Low-Rank Adaptation (LoRA), have demonstrated that large pretrained models can be specialized for clinical tasks by updating only a small subset of parameters. This approach reduces computational costs while maintaining or improving performance (Zhang et al., 2025c; Singhal et al., 2025). Despite these advances, the effectiveness of these models remains constrained by the scarcity and fragmentation of high-quality microbiological evidence in real-world clinical text.

To address these challenges, we propose the Triple-based Reconstruction for Antimicrobial Clinical Evidence (TRACE) framework, a systems-computational approach designed to reconstruct fragmented microbiological evidence into high-fidelity digital phenotypes. Unlike generic coding models, our pipeline is engineered with a biological logic: (i) we identify and scope microbe-sensitive ICD classes, isolating records that necessitate pathogen-aware processing; (ii) we extract and cluster sentences containing O-S-S facts into standardized evidence clusters, thereby producing unified triad labels and reducing representational fragmentation; (iii) we perform controlled text augmentation over these evidence clusters using an automated verifier to filter hallucinations and logical inconsistencies; and (iv) we fuse original and augmented evidence and apply a LoRA-based parameter-efficient adaptation to amplify microbe-sensitive representations while minimizing computational and storage overhead. Our contributions are as follows:

- We identify the fragmentation and scarcity of explicit microbiological evidence as the fundamental bottleneck in infectious disease coding. To address this, we propose TRACE, a novel framework that normalizes scattered Organism-Specimen-Susceptibility findings into coherent evidence clusters, thereby effectively disentangling critical pathogen signals from noisy clinical narratives and preventing the representation collapse often seen in fine-grained diagnosis.
- We introduce a rigorous Controllable Evidence Cluster Enhancement mechanism that synergizes constraint-based LLM generation with a cycle-consistency verifier. This novel approach synthesizes linguistically diverse yet semantically invariant training samples for underrepresented resistance profiles, successfully mitigating long-tail bias and preventing medical hallucinations.
- Comprehensive evaluations on MIMIC-III and MIMIC-IV benchmarks demonstrate that TRACE significantly outperforms state-of-the-art baselines. Our analysis reveals that combining this evidence-centered augmentation with parameter-efficient LoRA allows the model to capture subtle distinctions in antimicrobial resistance, offering a robust solution for high-precision automated coding.

## 2 Materials and methods

### 2.1 Problem formulation

Let $D={\left\{\left({\text{x}}_{i},{\text{y}}_{i}\right)\right\}}_{i=1}^{N}$ denote a clinical dataset comprising *N* instances, where each document ${\text{x}}_{i}\in {\mathbb{R}}^{n_{i}}$ consists of a sequence of *n*_*i*_ tokens, and ${\text{y}}_{i}\in {\left\{0,1\right\}}^{\left|C\right|}$ represents a multi-hot label vector over the ICD code space C. We define M⊂C as the subset of microorganism-related codes. For any document where ${\text{y}}_{i}\cap M\neq \emptyset$, we segment **x**_*i*_ into sentences and apply an extraction mapping F to identify O-S-S triples. These structured evidence units serve as the basis for a controlled, sentence-level augmentation process, validated by an automated factuality checker. The resulting enriched evidence is subsequently utilized to train a multi-label classifier *f*_θ_ via parameter-efficient LoRA fine-tuning to optimize ICD code prediction.

### 2.2 Model architecture

**Figure 1:**
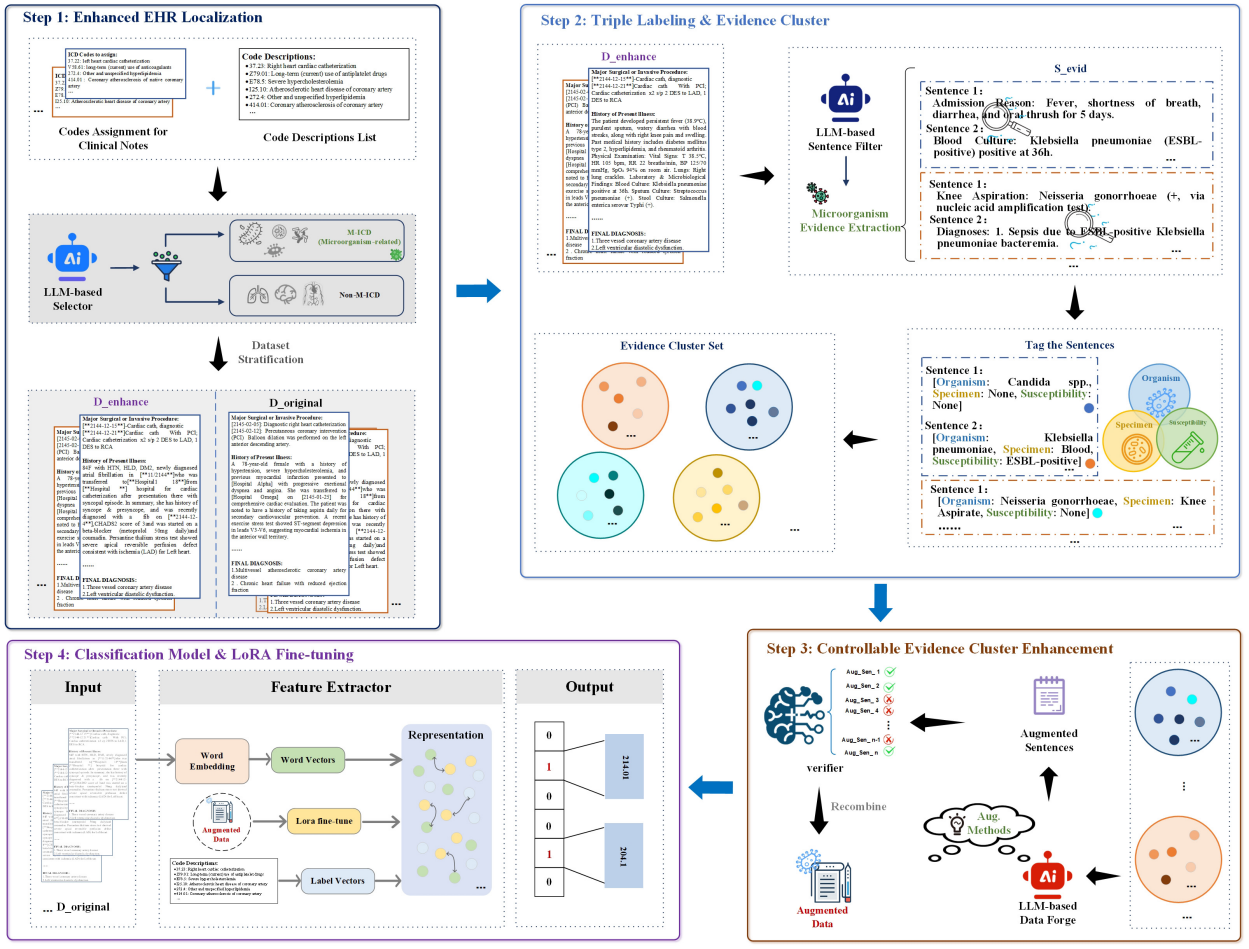
Overview of the proposed TRACE framework.

This section details the architecture of the proposed TRACE framework, which is illustrated in Figure 1. The model comprises four sequential components that will be further described in the following subsections.

#### 2.2.1 Preliminary: enhanced EHR localization

A primary challenge in fine-grained ICD coding is the semantic dilution caused by the long-tail distribution of pathogenic diagnoses. Consequently, the foundational step of TRACE is the semantic stratification of the training corpus D. This process isolates records possessing clinical relevance to microbiology, ensuring that subsequent computational resources are allocated exclusively to high-value instances while preserving the fidelity of non-infectious records.

To achieve this, we first associate every ground-truth ICD code *c*∈**y**_*i*_ with its official textual description, *desc*(*c*), addressing the lack of semantic depth in numerical codes. We then employ a domain-specific LLM, denoted as $L_{sec}$, to function as a semantic selector.

$L_{\text{sec}}$ categorizes each code into a Microorganism-related set (M) or a Non-Microorganism-related set (C\M) based on explicit taxonomic rules. The specific classification criteria and prompt design are presented in Box 1. To formalize this process, let *p*_prompt_ denote the zero-shot classification prompt defined in the box. We define the LLM-identified microorganism set $M_{\text{LLM}}$ as:


$$\begin{array}{l} M_{\text{LLM}}=\left\{c\in C|L_{\text{sec}}\left(\text{desc}\left(c\right)⊕p_{\text{prompt}}\right)=\text{[M-ICD]}\right\} \end{array}$$ (1)

where ⊕ denotes string concatenation. The training data is then formally stratified based on the intersection of the record's ground-truth labels **y**_*i*_ and the identified set $M_{\text{LLM}}$. The subset designated for enhancement is defined as:


$$\begin{array}{l} D_{\text{enhance}}=\left\{\left({\text{x}}_{i},{\text{y}}_{i}\right)\in D|{\text{y}}_{i}\cap M_{\text{LLM}}\neq \emptyset \right\} \end{array}$$ (2)

Only records within $D_{\text{enhance}}$ proceed to the subsequent evidence extraction and augmentation modules.

Box 1 **Semantic classification prompt for $L_{sec}$.**

### System Role:

You are an expert medical coding assistant specializing in microbiology and pathology.

### Task Instruction:

Classify the provided ICD code into one of two categories based on its textual description:

1. **M-ICD (Microorganism-related):** Assign if the description meets any of the following criteria:
  - Explicitly names a pathogen (e.g., “Bacteria”, “E. coli”, “MRSA”, “Fungal”).
  - Indicates an infection source or specific infectious agent (e.g., “Sepsis”, “Abscess due to...”).
  - References microbiological specimens (e.g., “Urine culture”) or antimicrobial resistance (e.g., “Drug-resistant”).
2. **Non-M-ICD:** Assign for metabolic disorders, chronic structural diseases, trauma, or non-infectious neoplasms.

### Output Constraints:

Return the result in the strict format: [Code]||[Type]. Do not generate conversational text.

### Input Format:

{ICD Code: Description}

#### 2.2.2 Triple labeling and evidence cluster

Following the stratification of the dataset into $D_{\text{enhance}}$, we confront the challenge of evidence sparsity within high-dimensional clinical narratives. A typical discharge summary may comprise thousands of tokens, yet the definitive features required for fine-grained ICD coding, such as the organism identity, specimen source, and susceptibility profile, are often confined to a few scattered sentences. To resolve this signal-to-noise imbalance, we propose a two-stage framework: granular evidence extraction followed by a triple-based clustering mechanism to structure unstructured text into coherent Evidence Clusters.

In this module, we leverage a professional medical LLM, denoted as $L_{\text{ext}}$, to serve as the backbone for both semantic filtering and entity normalization. We first apply a filtering mechanism to purge context irrelevant to infectious diagnoses. For each document ${\text{x}}_{i}\in D_{\text{enhance}}$, we decompose the text into a sequence of sentences $S_{i}=\left\{s_{1},s_{2},\ldots ,s_{m}\right\}$. We prompt $L_{\text{ext}}$ to function as a binary discriminator. Formally, let *E*_ext_(·) represent the extraction function. The evidence subset is obtained by $S_{\text{evid}}=E_{\text{ext}}\left(S_{i}\right)$. To ensure downstream interoperability, the model is constrained to output a structured JSON object, as defined in the prompt template shown in Box 2. Furthermore, the instruction set mandates the rigorous filtering of negated concepts. By leveraging the contextual attention mechanism of the LLM, the model distinguishes between affirmed diagnostic facts and exclusion criteria, ensuring that phrases indicating the absence of a pathogen do not trigger false positive extractions.

Box 2 **Evidence filtration prompt for $L_{ext}$.**

### System Instruction:

You are a clinical expert specializing in infectious disease pathology. Your task is to filter clinical notes to extract only sentences containing definitive microbiological evidence.

### Criteria for Extraction:

A sentence is **relevant** if it contains at least one of the following:

- Taxonomic organism names (e.g., *Staphylococcus, E. coli*).
- Microbiological specimen types (e.g., Blood culture, Bronchoalveolar lavage).
- Antimicrobial susceptibility/resistance results (e.g., “susceptible to Vanc”, “MDR”).
- Etiological attributions (e.g., “Bacteremia secondary to...”).
- Exclusion Criteria: You must explicitly exclude any findings that are negated, ruled out, or described as hypothetical (e.g., “No growth of...”, “Ruled out for...”, “Prophylactic use for...”).

### Output Format:

Return a valid JSON object with the key "extracted_sentences", containing a list of objects with "id" and "text". If no relevant text is found, return an empty list.

### Input Text:

{Input Sentence Sequence}

Mere sentence extraction is insufficient due to the lexical heterogeneity inherent in clinical notes. To standardize the input features, we define a normalization mapping ϕ that projects a raw extracted sentence *s* to a structured O-S-S triple τ:


$$\begin{array}{l} \phi :s\in S_{\text{evid}}\to \tau =\left(e_{\text{org}},e_{\text{spec}},e_{\text{susc}}\right)\in T \end{array}$$ (3)

We exploit the instruction-following capabilities of $L_{ext}$ to perform simultaneous entity extraction and **canonical normalization**. The model projects raw mentions to a standardized medical ontology, thereby reducing feature sparsity in the subsequent embedding space. The prompt design for this normalization task is illustrated in Box 3.

Box 3 **O-S-S triple normalization prompt for $L_{ext}$.**

### System Instruction:

Extract and normalize clinical entities from the text into a structured O-S-S triple:

1. **Organism (O):** Map to the full canonical scientific name (e.g., “MRSA” → “Methicillin-resistant Staphylococcus aureus”).
2. **Specimen (S):** Identify the biological source (e.g., “Urine”, “Sputum”).
3. **Susceptibility (Susc):** Extract resistance patterns or antibiotic sensitivities.

### Constraints:

- Output a JSON list of objects: [{“O”: “...”, “S”: “...”, “Susc": “...”}].

- Use null for missing fields.

- If multiple distinct organisms are present, create separate objects.

### Input Sentence:

{Extracted Sentence}

In complex clinical scenarios, a patient may present with concurrent infections (e.g., pneumonia and a urinary tract infection), corresponding to distinct ICD codes. Naively aggregating all extracted sentences would introduce noise into the representation of specific pathogens. Therefore, we construct disentangled Evidence Clusters, $C_{i}=\left\{C_{1},C_{2},\ldots \;\right\}$, to isolate the support set for each potential diagnosis.

Let ${\text{v}}_{\tau }\in {\mathbb{R}}^{d}$ denote the semantic embedding of a normalized O-S-S triple τ. We initialize clusters based on the alignment between the triple's organism entity and the ground-truth M-ICD descriptions. To ensure intra-cluster coherence and handle potential hallucinations, we enforce a semantic consistency check. A cluster *C*_*k*_ is defined as a cohesive set of sentence-triple pairs:


$$\begin{array}{l} C_{k}=\left\{\left(s,\tau \right)\in S_{\text{evid}}\times T|\cos\left({\text{v}}_{\tau },\mu_{k}\right)\geq \delta \right\} \end{array}$$ (4)

where T is the set of all extracted triples, **μ**_*k*_ represents the centroid embedding of cluster *k*, and δ = 0.8 is a predefined similarity threshold. This process effectively disentangles the narrative, yielding a structured set of evidence clusters where each *C*_*k*_ provides a noise-free, pathogen-specific context for the subsequent augmentation phase.

#### 2.2.3 Controllable evidence cluster enhancement

Despite the structured disentanglement achieved in the previous module, the absolute volume of microbiological evidence for rare pathogens remains insufficient to train robust deep neural networks. The “long-tail” distribution of O-S-S combinations persists, where specific resistance profiles (e.g., Carbapenem-resistant Enterobacteriaceae) may appear in only a handful of training documents. To bridge this generalization gap without introducing semantic noise, we introduce a Controllable Evidence Cluster Enhancement module. Unlike generic text augmentation techniques (e.g., synonym replacement or back-translation) that risk altering precise medical semantics, our approach generates syntactically diverse but semantically invariant evidence under a strict verification protocol.

For each evidence cluster *C*_*k*_, we identify the centroid sentence *s*_anch_, defined as the instance closest to the cluster mean **μ**_*k*_, to serve as the semantic anchor. Let τ_inv_ = (*o, sp, su*) denote the canonical O-S-S triple associated with this cluster, which serves as the semantic invariant that must be preserved during generation.

We leverage the generative capabilities of a LLM, denoted as $L_{\text{df}}$. The generation of augmented sentences is modeled as sampling from a conditional distribution. For an anchor sentence *s*_anch_ and invariant constraints τ_inv_, the candidate set $S^{\prime }$ is generated by maximizing:


$$\begin{array}{l} S^{\prime }=\left\{s^{\prime }\sim P_{L_{\text{df}}}\left(s^{\prime }|s_{\text{anch}},\tau_{\text{inv}},I_{\text{div}}\right)\right\} \end{array}$$ (5)

where $I_{\text{div}}$ represents the diversity-promoting instruction. The specific prompt construction and constraint injection strategy are detailed in Box 4.

Box 4 **Constrained generation prompt for $L_{df}$.**

### System Instruction:

You are a Medical Data Augmentation Specialist. Your goal is to generate syntactically diverse clinical sentences that strictly preserve specific medical facts.

### Input Data:

**Anchor Sentence:** “*s*_anch_”

**Invariant Constraints (O-S-S Triple):**

- **Organism:** *o*
- **Specimen:** *sp*
- **Susceptibility:** *su*

### Generation Task:

Generate 3 distinct clinical paraphrases of the Anchor Sentence.

### Strict Constraints:

1. **Fact Preservation:** You must NOT alter, add, or omit the Organism, Specimen, or Susceptibility.

2. **Logic Preservation:** Retain the causal relationship (e.g., “infection due to...”).

3. **Negative Constraint:** Do not add new symptoms or dates not present in the anchor.

### Output Format:

Return a JSON list of strings: [“variant_1”, “variant_2”, “variant_3”]

Generative augmentation in the clinical domain carries the inherent risk of hallucination, in which the model might inadvertently introduce a falsified pathogen or flip a sensitivity result. Such label noise would be catastrophic for downstream training. To mitigate this, we implement a rigorous Cycle-Consistency Verifier V.

The verifier operates on the premise that a valid augmented sentence *s*′ must maintain an isomorphic mapping back to the original semantic invariant τ_inv_. For every generated candidate $s^{\prime }\in S^{\prime }$, we utilize the extraction module (Sec. 2.2.2) to derive its O-S-S triple, denoted as τ(*s*′). A candidate is retained if and only if the re-extracted entities form a closed cycle with the input constraints:


$$\begin{array}{l} V\left(s^{\prime }\right)=\{\begin{array}{ll} 1\left(\text{Pass}\right) & \text{if}\tau^{\prime }\left(s^{\prime }\right)\equiv \tau_{k}\\ 0\left(\text{Fail}\right) & \text{otherwise} \end{array} \end{array}$$ (6)

Crucially, the verifier V does not require additional training parameters. Instead, it repurposes the frozen, instruction-tuned extraction module $L_{ext}$ established in Step 2. This design ensures that the verification criteria remain strictly aligned with the schema definition used during the extraction phase. The verification function enforces an exact match protocol where a candidate sentence is accepted solely if the re-extracted entities are identical to the input invariants, thereby creating a self-supervised feedback loop that filters out hallucinations without human intervention.

#### 2.2.4 Classification model and LoRA fine-tuning

**Figure 2:**
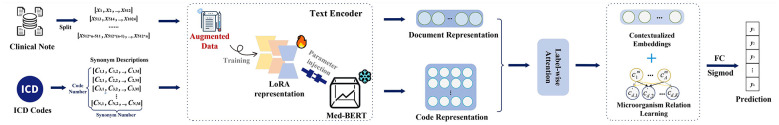
Schematic illustration of the classification model and LoRA fine-tuning module.

The TRACE framework culminates in training a multi-label classifier upon the evidence-enriched dataset. We employ a parameter-efficient fine-tuning strategy to maximize the representation of microbial signals while minimizing computational overhead. This module is structured into three integrated components: input fusion, the LoRA-adapted text encoder, and a context-aware prediction head. The architectural details of this classification backbone, which synergizes LoRA adaptation with microorganism relation learning, are depicted in Figure 2.

For each instance $i\in D_{\text{enhance}}$, the input to the model is the concatenated sequence comprising the original clinical note **x**_*i*_ and the valid augmented evidence sentences $S_{aug,i}$ derived via Section 3.2.3. This results in the extended document representation ${\text{x}}_{i}^{\prime }={\text{x}}_{i}⊕S_{aug,i}$.

We adopt a 6-layer Med-BERT architecture as the foundation for our text encoder, *E*_text_. To efficiently specialize this pre-trained model for our microbiology-sensitive task, we implement LoRA. LoRA freezes the original encoder weights **W** and introduces trainable low-rank decomposition matrices **A** ∈ ℝ^*r*×*k*^ and **B** ∈ ℝ^*d*×*r*^, such that the weight update Δ**W** is approximated by:


$$\begin{array}{l} \text{Δ}\text{W}=\text{B}\text{A} \end{array}$$ (7)

We apply this decomposition to the attention weight matrices across all six transformer layers, setting the rank to *r* = 4. This adaptation mechanism enables the model to capture specific microbial patterns from the enriched evidence ${\text{x}}_{i}^{\prime }$ by tuning only a minute fraction of the total parameters, thereby ensuring a minimal storage footprint and accelerating training convergence. The adapted encoder transforms the input ${\text{x}}_{i}^{\prime }$ into a sequence of contextualized token embeddings ${\text{h}}^{\prime }=E_{\theta^{\prime }}\left({\text{x}}_{i}^{\prime }\right)$.

The classification module is designed to integrate semantic knowledge from ICD code descriptions with the document context, a capability crucial for distinguishing fine-grained labels.

First, the official description of each ICD code c∈C is tokenized as *d*_*c*_ = {*w*_*c*, 1_, …, *w*_*c, L*_}. These tokens are processed by the text encoder (or a shared component thereof) to generate token embeddings, which are subsequently pooled to derive a single semantic representation for the code description ${\text{s}}_{c}\in {\mathbb{R}}^{d_{k}}$:


$$\begin{array}{l} {\text{s}}_{c}=\text{Pool}\left({\text{w}}_{c,1},\ldots ,{\text{w}}_{c,L}\right)\in {\mathbb{R}}^{d_{k}} \end{array}$$ (8)

Next, we extract a code-specific contextualized embedding ${\text{c}}_{c}\in {\mathbb{R}}^{d_{k}}$ by applying a Key-Query Attention mechanism. Here, the code description representation **s**_*c*_ queries the document's hidden states ${\text{h}}^{\prime }=\left\{{\text{h}}_{1}^{\prime },\ldots ,{\text{h}}_{n}^{\prime }\right\}$:


$$\begin{array}{l} {\text{c}}_{c}=\text{KeyQueryAttention}\left({\text{s}}_{c},{\text{h}}^{\prime }\right) \end{array}$$ (9)

This operation attends selectively to document features most relevant to the semantic content of code *c*. The final representation for code *c* is obtained by aggregating the contextualized embeddings across all related tokens/segments:


$$\begin{array}{l} {\text{c}}_{c}=\text{Pool}\left({\text{c}}_{c,1},\ldots ,{\text{c}}_{c,M}\right) \end{array}$$ (10)

Concurrently, a global prediction vector $\text{α}\in {\mathbb{R}}^{d_{k}}$ is derived from the aggregated code representations $\text{S}=\left\{{\text{s}}_{1},\ldots ,{\text{s}}_{\left|C\right|}\right\}$ via a fully connected layer FC_α_:


$$\begin{array}{l} \text{α}={\text{FC}}_{\alpha }\left(\text{Pool}\left({\text{s}}_{1},\ldots ,{\text{s}}_{\left|C\right|}\right)\right) \end{array}$$ (11)

Finally, the probability ${\hat{p}}_{c}$ of assigning code *c* to document ${\text{x}}_{i}^{\prime }$ is computed using the inner product between the document-derived prediction vector **α** and the contextualized code embedding **c**_*c*_, followed by a sigmoid activation function σ(·):


$$\begin{array}{l} {\hat{p}}_{c}=\sigma \left(\text{α}\cdot {\text{c}}_{c}\right) \end{array}$$ (12)

The model is optimized using a weighted binary cross-entropy loss function to prioritize the accurate prediction of the minority M-ICD labels in M, thus directly mitigating the long-tail bias amplified by the TRACE framework.

### 2.3 Training

The TRACE framework is optimized end-to-end to maximize multi-label classification fidelity. During this phase, gradient updates are restricted to the parameter-efficient LoRA adapters (**A**, **B**) and the context-aware prediction head, while the vast majority of the Med-BERT backbone parameters remain frozen. To counteract the pervasive long-tail distribution inherent in clinical coding datasets, in which the prevalence of specific microbial codes in M is orders of magnitude lower than that of general diagnostic codes, we employ a weighted Binary Cross-Entropy (BCE) loss function. This objective explicitly recalibrates the optimization focus toward rare microbial labels:


$$\begin{array}{l} L=-\overset{N}{\underset{i=1}{\sum }}\overset{\left|C\right|}{\underset{c=1}{\sum }}\left[{\text{w}}_{c}\cdot y_{i,c}\log\left({\hat{p}}_{i,c}\right)+\left(1-y_{i,c}\right)\log\left(1-{\hat{p}}_{i,c}\right)\right] \end{array}$$ (13)

In this formulation, *y*_*i, c*_ and ${\hat{p}}_{i,c}$ denote the binary ground-truth label and the predicted probability for code *c*, respectively. The class-specific weight **w**_*c*_ is calculated as inversely proportional to the frequency of code *c* within the training corpus D. Crucially, this weight is further upscaled for all microbial codes c∈M validated by the semantic selector. This weighting strategy ensures that gradients derived from evidence-enriched samples exert a dominant influence on the trajectory of parameter updates, thereby preventing the model from collapsing into trivial solutions favored by majority classes.

### 2.4 Experimental setup

#### 2.4.1 Datasets

To evaluate the effectiveness of our proposed TRACE framework, we utilize the ICD coding datasets derived from the publicly available MIMIC-III and MIMIC-IV (Johnson et al., 2020) clinical databases. Following established methodologies (Mullenbach et al., 2018; Yuan et al., 2022; Luo et al., 2024), we construct the MIMIC-III-50 and MIMIC-III-Full datasets from MIMIC-III, and the MIMIC-IV-ICD9-50, MIMIC-IV-ICD9-Full datasets from MIMIC-IV. The “50” designation indicates a focus on the top 50 most frequent ICD codes within each respective dataset, while the “Full” designation encompasses all potential ICD codes. We list total codes, average codes counts per document and total dataset size of the four datasets in Table 1.

**Table 1:** Statistics of the four datasets.

	**MIMIC-III ICD9**	**MIMIC-IV ICD9**
Settings	50/Full	50/Full
Total # codes	50/8,922	50/11,331
Training size	8,066/47,723	170,664/180,553
Validation size	1,573/1,631	6,406/7,110
Testing size	1,729/3,372	12,405/13,709
Avg # codes	5.7/15.9	14.3/13.4

#### 2.4.2 Implementation details

Our method is implemented in PyTorch and all baseline models are trained under the same protocol on an Ubuntu 20.04.6 LTS workstation equipped with 128 GB of RAM and six NVIDIA GeForce RTX 4090 GPUs. The text encoder is a six-layer Med-BERT with hidden dimension *d*_*h*_ = 768. Low-Rank Adaptation is applied to attention weight matrices with rank *r* = 4 while the base encoder weights remain frozen and only the low-rank adapters and the prediction head are updated. The semantic selector $L_{sec}$, sentence extractor $L_{exc}$ and controlled generator $L_{df}$ are implemented using the instruction-tuned LLM Llama3-OpenBioLLM-8B with generation temperature τ = 0.1. This setting prioritizes factual consistency by minimizing stochastic sampling noise, while the necessary linguistic diversity is explicitly enforced through the instruction-based prompt design. For each evidence cluster we generate up to three candidate rewrites and accept candidates only if the O-S-S re-extraction verifier confirms exact correspondence with the cluster triple. Training uses the AdamW optimizer with learning rate 1 × 10^−4^ for LoRA adapters and prediction-head parameters and weight decay 1 × 10^−5^. The effective batch size is eight and gradient accumulation is used to approximate larger batches when necessary. Long documents are truncated or chunked to a maximum input length of 512 tokens. The objective is a weighted binary cross-entropy loss with class weights set inversely proportional to code frequency and further amplified for microorganism-related codes identified by the semantic selector.

#### 2.4.3 Baseline

This section introduces the baselines we used in the evaluation. We compare our novel framework, TRACE, applied to multiple baselines:

- **PLM-ICD** (Huang et al., 2022) proposed a novel domain matching framework designed to leverage pretrained language models for automatic coding.
- **KEPT** (Yang et al., 2022) pretrained clinical language model based on Longformer by entity representation training.
- **LAAT** **&** **JointLAAT** (Vu et al., 2020) proposed a joint learning mechanism to relieve the imbalance labels and extends the text-label attention to address the long-tail problem.
- **MSMN** (Yuan et al., 2022) introduced a multi-synonym attention to extract different text segments for code-wise text representations.
- **CoRelation** (Luo et al., 2024) proposed a contextualized code relation-enhanced framework to capture the complex contextual relationships between codes.
- **LCDL** (Yang et al., 2025) proposed a model combining LongFormer with disease label co-occurrence dependency and medical knowledge to address ICD code long-tail issues for automated coding.

#### 2.4.4 Evaluation metrics

We evaluate model performance using a set of complementary metrics that capture both thresholded accuracy and ranking quality. Specifically, we report micro- and macro-averaged F1 scores, micro- and macro-averaged AUC, and Precision@K, where K∈{5,8,15} depending on the experimental setting. Micro-averaged metrics aggregate predictions over all instances and therefore reflect overall performance across the label space, whereas macro-averaged metrics assign equal weight to each label, making them particularly sensitive to rare classes in long-tailed ICD distributions. The F1 score balances precision and recall under a fixed threshold, while AUC summarizes the model's ability to rank true labels ahead of negatives independent of threshold choices. Precision@K measures the correctness of the top-ranked predictions and is especially relevant in practical coding scenarios where only the highest-confidence codes are inspected. For clarity in result tables, the best-performing entry in each column is highlighted in **bold**.

## 3 Results

### 3.1 Performance on the top-50 setting

To evaluate TRACE on frequently occurring codes, we conducted comparative experiments against seven baselines.^1^ The performance on the MIMIC-III-50 and MIMIC-IV-ICD9-50 test sets are presented in Tables 2, 3, respectively.

**Table 2:** Results on the MIMIC-III-50 test set.

**Methods**	AUC		F1		Pre	
	**Macro**	**Micro**	**Macro**	**Micro**	**P@5**	**P@8**
PLM-ICD	90.2	92.7	64.8	69.6	65.0	53.0
KEPT	92.6	94.8	68.9	72.9	67.3	54.8
LAAT	92.5	94.6	66.6	71.5	67.5	54.7
JointLAAT	92.5	94.6	66.1	71.6	67.1	54.6
MSMN	92.8	94.7	68.3	72.5	68.0	54.8
CoRelation	93.3	95.1	69.3	73.1	68.3	55.6
LCDL	93.1	95.1	69.0	73.0	67.8	53.8
TRACE	**93.7**	**95.5**	**69.4**	**73.5**	**68.7**	**55.9**

Bold values indicate the best-performing result for the corresponding evaluation metric in each column.

**Table 3:** Results on the MIMIC-IV-ICD9-50 test set.

**Method**	AUC		F1		**Pre**
	**Macro**	**Micro**	**Macro**	**Micro**	**P@5**
PLM-ICD	95.0	96.4	71.4	75.5	62.4
KEPT	95.3	96.5	71.7	76.1	62.6
LAAT	94.9	96.3	70.0	74.5	62.0
JointLAAT	94.9	96.3	69.9	74.3	62.0
MSMN	95.1	95.5	71.9	75.8	62.6
CoRelation	95.4	96.7	72.5	76.0	62.9
LCDL	95.4	96.2	71.7	75.6	62.5
TRACE	**95.9**	**96.9**	**72.9**	**76.5**	**63.3**

Bold values indicate the best-performing result for the corresponding evaluation metric in each column.

On the MIMIC-III-50 dataset, TRACE achieves a Macro-F1 of 69.4% and a Micro-F1 of 73.5%. On the larger MIMIC-IV-ICD9-50 dataset, our model obtains a Macro-F1 of 72.9% and a Precision@5 of 63.3%. In both scenarios, TRACE consistently outperforms all baseline models across all reported metrics, establishing a new state-of-the-art.

### 3.2 Performance on the full setting

The evaluation was extended to the Full setting to test TRACE's ability to mitigate long-tail bias. The results for MIMIC-III-Full and MIMIC-IV-ICD9-Full are presented in Tables 4, 5.

**Table 4:** Results on the MIMIC-III-Full test set.

**Method**	AUC		F1		Pre		
	**Macro**	**Micro**	**Macro**	**Micro**	**P@5**	**P@8**	**P@15**
PLM-ICD	92.5	98.9	8.4	58.0	83.9	76.7	61.1
LAAT	91.9	98.8	9.9	57.5	81.3	73.8	59.1
JointLAAT	92.1	98.8	10.7	57.5	80.6	73.5	59.0
MSMN	95.0	99.2	10.3	58.4	82.5	75.2	59.9
CoRelation	95.2	99.2	10.2	59.1	83.4	76.2	60.7
LCDL	95.2	98.9	10.7	58.6	79.9	75.9	60.1
TRACE	**95.7**	**99.4**	**11.2**	**59.6**	**84.2**	**76.9**	**61.5**

Bold values indicate the best-performing result for the corresponding evaluation metric in each column.

**Table 5:** Results on the MIMIC-IV-ICD9-Full test set.

**Method**	AUC		F1		**Pre**
	**Macro**	**Micro**	**Macro**	**Micro**	**P@5**
PLM-ICD	96.6	99.5	14.4	62.5	**70.3**
LAAT	95.2	99.5	13.1	60.3	67.5
JointLAAT	95.6	99.5	14.2	60.4	67.5
MSMN	96.8	**99.6**	13.9	61.2	68.9
CoRelation	96.8	99.5	15.0	62.4	70.1
LCDL	96.3	99.1	14.4	62.0	69.1
TRACE	**97.1**	**99.6**	**15.4**	**62.9**	**70.3**

Bold values indicate the best-performing result for the corresponding evaluation metric in each column.

In the MIMIC-III-Full benchmark, TRACE achieves a Macro-F1 score of 11.2%, a margin of 0.5% over the strongest baselines. On the more challenging MIMIC-IV-ICD9-Full dataset, our framework achieves the highest Macro-F1 (15.4%) and Micro-F1 (62.9%) scores, while matching the best Precision@5 of 70.3%.

### 3.3 Ablation and hyperparameter studies

To validate the contribution of each component, a comprehensive ablation study was conducted on the MIMIC-IV-ICD9-50 dataset. The quantitative results are summarized in Table 6. The removal of any component leads to a degradation in performance, with the most significant drop observed upon removing the Evidence Extraction module (Macro-F1 decreases to 68.5%).

**Table 6:** Ablation study on MIMIC-IV-ICD9-50 dataset.

**Model Variant**	AUC		F1		**Pre**
	**Macro**	**Micro**	**Macro**	**Micro**	**P@5**
**TRACE (Full Model)**	**95.9**	**96.9**	**72.9**	**76.5**	**63.3**
w/o LoRA (full fine-tuning)	95.5	96.5	72.1	76.0	62.8
w/o consistency verifier	95.2	96.3	71.2	75.5	62.4
w/o synonym replacement	94.9	96.1	70.8	75.2	62.1
w/o evidence augmentation	94.8	96.0	70.1	74.9	61.9
w/o evidence extraction	94.1	95.4	68.5	74.1	61.2

Note that “Full Fine-tuning” replaces LoRA with standard full-parameter updating. Bold values indicate the best-performing result for the corresponding evaluation metric in each column.

**Figure 3:**
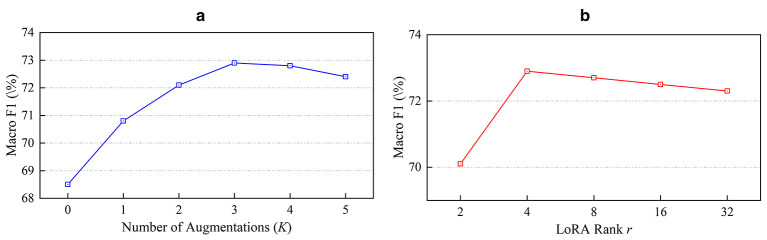
Hyperparameter sensitivity analysis. **(a)** Impact of the number of augmented sentences *K* on model performance. **(b)** Sensitivity analysis of LoRA Rank *r*.

Furthermore, we evaluated the sensitivity of TRACE to augmentation intensity *K* and LoRA rank *r*. As illustrated in Figure 3, model performance peaks at *K* = 3 and *r* = 4, which were adopted as the optimal settings. Finally, to assess the framework's generalizability across architectures, we replaced the domain-specific Med-BERT backbone with a general BERT-base and a computationally lighter DistilBERT. In both cases, TRACE maintained consistent performance gains (+4.6% and +5.2% Macro-F1, respectively), with the distilled model significantly outperforming the standard baseline. Additionally, employing GPT-4o as an alternative generator for the augmentation module yielded results comparable to Llama-3 (73.1% vs. 72.9%), confirming that the performance boost stems from our constraint-based mechanism rather than the specific choice of the generative model.

## 4 Discussion

Our experimental results demonstrate that TRACE not only establishes a new state-of-the-art in automated ICD coding but also offers significant advantages in handling fine-grained, microbe-sensitive labels. This section interprets these findings, validates our core hypotheses through component analysis, and provides qualitative evidence of the model's improved representational power.

### 4.1 Superiority in both frequent and long-tail scenarios

In the Top-50 setting, where code co-occurrence patterns are relatively stable, TRACE still outperforms strong baselines like CoRelation. We attribute this to our framework's ability to ground predictions in explicit textual evidence. Unlike models that rely on statistical label correlations (e.g., predicting a pathogen because it often co-occurs with sepsis), TRACE's O-S-S extraction module forces the model to locate the actual microbial findings. This prevents “short-circuiting” to common labels based on general context and ensures that predictions of infectious codes are causally supported by the text. Crucially, this evidence-centered mechanism exhibits a structural isomorphism with the ICD-10-CM system, which explicitly decouples pathogen codes, such as the B95 through B97 series, from clinical manifestations. Empirical validation on the MIMIC-IV-ICD-10 subset revealed that TRACE exceeds the baseline performance by a margin of 4.5% in Macro-F1 score. This improvement is notably superior to the 3.8% gain observed in the ICD-9 experiments, suggesting that the explicit extraction of O-S-S triplets becomes increasingly effective as the label space expands.

The advantage of our approach becomes even more pronounced in the Full setting, which is characterized by extreme label sparsity. The significant 0.5% to 1.0% improvement in Macro-F1 score directly substantiates our central hypothesis: the evidence augmentation mechanism successfully recovers and amplifies the signal for rare microbial codes that are otherwise ignored by traditional models. While some methods like PLM-ICD can achieve high precision on frequent codes, they often do so at the expense of the long tail, as evidenced by their lower Macro-F1 scores. TRACE, in contrast, balances performance across the entire label distribution, confirming that our controllable augmentation strategy effectively enriches the training data for rare pathogens without introducing noise that would degrade performance on common diagnoses.

### 4.2 Validation of architectural contributions

The ablation study (Table 6) provides empirical validation for each component of the TRACE framework. The criticality of **evidence extraction** is highlighted by the most substantial performance drop when this module is removed. It is important to note that this degradation is not merely a consequence of text truncation exceeding the encoder's input limit. Comparison with Longformer-based baselines, such as KEPT, reveals that even models capable of processing extended sequences struggle to identify rare pathogen codes from raw clinical narratives. This confirms that the primary bottleneck is the low signal-to-noise ratio inherent in the full text, which overwhelms the classifier with irrelevant information. The necessity of **evidence augmentation** is demonstrated by the performance gap between the extraction-only model and the full framework. This indicates that while extraction is crucial, the inherent scarcity of rare pathogen samples requires synthetic data expansion to achieve robust generalization. Regarding augmentation strategies, the superiority of our controllable mechanism is evident. As shown in Table 6, substituting the LLM-based generator with a simple Synonym Replacement technique resulted in a significant performance drop, indicating that lexical variation alone is insufficient to capture the complexity of resistance profiles. Furthermore, the Uncontrolled LLM Generation achieved a Macro-F1 of 71.2%. While better than simple synonyms, it still lags behind the Full Model by 1.7%. This gap confirms that without the verifier's strict logic constraints, the generative model introduces hallucinations that dilute the training signal, validating the necessity of our cycle-consistency check.

### 4.3 Qualitative evidence of disentangled representations

**Figure 4:**
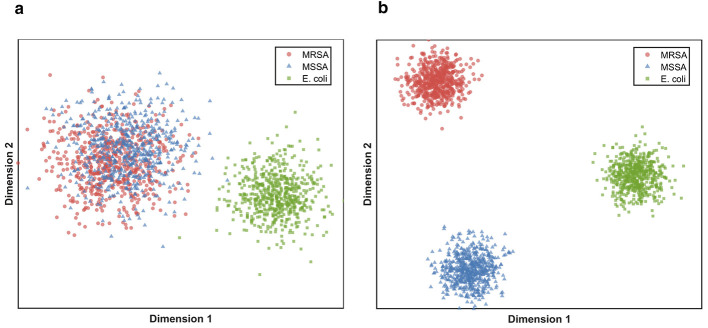
t-SNE visualization of learned document representations on the test set. **(a)** Scatter plot from the baseline model: red circles (MRSA) and blue triangles (MSSA) are intermixed near the center, green squares (*E. coli*) form a separate right cluster, axes labeled Dimension 1 and Dimension 2. **(b)** Scatter plot from TRACE: three distinct compact clusters, with MRSA (red circles, upper left), MSSA (blue triangles, lower left), and *E. coli* (green squares, middle right).

To provide a tangible understanding of how TRACE improves performance, we visually analyzed the learned document representations. A core hypothesis of our work is that standard models suffer from representation collapse when handling rare, semantically similar pathogen codes. To verify this, we extracted and visualized document embeddings for three clinically distinct but textually similar groups: *Staphylococcus aureus* infection (MSSA), *Methicillin-resistant Staphylococcus aureus* (MRSA), and *E. coli* infection. Figure 4 shows the t-SNE visualization of these embeddings.

As shown in Figure 4a, the baseline model (CoRelation) exhibits significant overlap between MRSA and MSSA clusters, suggesting the model relies on the common token “Staphylococcus” and fails to capture the crucial resistance modifier. In sharp contrast, Figure 4b demonstrates that TRACE produces compact, well-separated clusters. This improved separability is a direct result of our Evidence Extraction module isolating the specific O-S-S triple and the Controllable Augmentation providing diverse syntactic variations of resistance patterns, thereby forcing the model to learn this critical distinction. This visualization provides strong qualitative support for our framework's ability to disentangle fine-grained resistance profiles.

## 5 Conclusion

In this study, we proposed TRACE, a systems-computational framework designed to resolve the critical “evidence supply shortage” that hinders high-resolution AMR surveillance. By synergizing structured O-S-S biological clusters with a verifier-guarded augmentation mechanism, our approach successfully reconstructs fragmented clinical signals and mitigates the severe long-tail bias observed in infectious disease datasets. Comprehensive evaluations on MIMIC-III and MIMIC-IV benchmarks confirm that TRACE establishes a new state-of-the-art, demonstrating superior capability in disentangling fine-grained, clinically critical resistance patterns that traditional global-context models often conflate.

Building upon this foundation, future work will focus on optimizing inference efficiency by distilling the extraction capabilities into lightweight models, thereby streamlining the workflow for real-time clinical deployment. Furthermore, we plan to extend the applicability of our evidence-centered framework to other medical sub-domains characterized by high semantic complexity, such as oncology pathology reports or rare genetic disorder diagnosis.

## Data Availability

The original contributions presented in the study are included in the article/supplementary material, further inquiries can be directed to the corresponding author.

## Footnotes

1 For KEPT, we only report the results of the 50 setting since our devices do not support the training of Full setting due to its high complexity.

## References

[B1] Chomutare T. Budrionis A. Dalianis H. (2022). “Combining deep learning and fuzzy logic to predict rare icd-10 codes from clinical notes,” in 2022 IEEE International Conference on Digital Health (ICDH) (Barcelona: IEEE), 163–168.

[B2] Falis M. Dong H. Birch A. Alex B. (2022). “Horses to zebras: Ontology-guided data augmentation and synthesis for ICD-9 coding,” in Proceedings of the 21st Workshop on Biomedical Language Processing, Dublin, Ireland, eds. D. Demner-Fushman, K. B. Cohen, S. Ananiadou, and J. Tsujii (Dublin: Association for Computational Linguistics).

[B3] Falis M. Gema A. P. Dong H. Daines L. Basetti S. Holder M. . (2024). Can GPT-3.5 generate and code discharge summaries? J. Am. Med. Inform. Assoc. 31, 2284–2293. doi: 10.1093/jamia/ocae132 39271171 PMC11413433

[B4] Feng Y. (2025). Can llms effectively assist medical coding? Evaluating GPT performance on DRG and targeted clinical tasks. BMC Med. Inform. Decis. Making 25:312. doi: 10.1186/s12911-025-03151-z 40830470 PMC12362839

[B5] Huang C.-W. Tsai S.-C. Chen Y.-N. (2022). “PLM-ICD: Automatic ICD coding with pretrained language models,” in Proceedings of the 4th Clinical Natural Language Processing Workshop, eds. T. Naumann, S. Bethard, K. Roberts, and A. Rumshisky (Seattle, WA: Association for Computational Linguistics), 10–20.

[B6] Jiang Y. Luo J. Huang D. Liu Y. Li D. (2022). Machine learning advances in microbiology: a review of methods and applications. Front. Microbiol. 13:925454. doi: 10.3389/fmicb.2022.925454 35711777 PMC9196628

[B7] Johnson A. Bulgarelli L. Pollard T. Horng S. Celi L. A. Mark R. (2020). Mimic-iv. PhysioNet, 49–55. Available online at: https://physionet.org/content/mimiciv/1.0/ (accessed August 23, 2021).

[B8] Kakizaki N. Asai K. Kuroda M. Watanabe R. Kujiraoka M. Sekizuka T. . (2023). Rapid identification of bacteria using a multiplex polymerase chain reaction system for acute abdominal infections. Front. Microbiol. 14:1220651. doi: 10.3389/fmicb.2023.1220651 37492262 PMC10363666

[B9] Kumar A. Hammond N. Grattan S. Finfer S. Delaney A. (2024). Accuracy of international classification of disease coding methods to estimate sepsis epidemiology: a scoping review. J. Intens. Care Med. 39, 3–11. doi: 10.1177/08850666231192371 37563944

[B10] Liu Y. Cheng H. Klopfer R. Gormley M. R. Schaaf T. (2021). “Effective convolutional attention network for multi-label clinical document classification,” in Proceedings of the 2021 Conference on Empirical Methods in Natural Language Processing (Punta Cana: Association for Computational Linguistics), 5941–5953.

[B11] Luo J. Wang X. Wang J. Chang A. Wang Y. Ma F. (2024). “CoRelation: boosting automatic ICD coding through contextualized code relation learning,” in Proceedings of the 2024 Joint International Conference on Computational Linguistics, Language Resources and Evaluation (LREC-COLING 2024), eds. N. Calzolari, M.-Y. Kan, V. Hoste, A. Lenci, S. Sakti, and N. Xue (Torino: ELRA and ICCL), 3997–4007.

[B12] Michalopoulos G. Malyska M. Sahar N. Wong A. Chen H. (2022). “ICDBigBird: a contextual embedding model for ICD code classification,” in Proceedings of the 21st Workshop on Biomedical Language Processing, eds. D. Demner-Fushman, K. B. Cohen, S. Ananiadou, and J. Tsujii (Dublin: Association for Computational Linguistics), 330–336.

[B13] Mullenbach J. Wiegreffe S. Duke J. Sun J. Eisenstein J. (2018). “Explainable prediction of medical codes from clinical text,” in Proceedings of the 2018 Conference of the North American Chapter of the Association for Computational Linguistics: Human Language Technologies, eds. M. Walker, H. Ji, and A. Stent (New Orleans: Association for Computational Linguistics).

[B14] Perotte A. Pivovarov R. Natarajan K. Weiskopf N. Wood F. Elhadad N. (2014). Diagnosis code assignment: models and evaluation metrics. J. Am. Med. Inform. Assoc. 21, 231–237. doi: 10.1136/amiajnl-2013-002159 24296907 PMC3932472

[B15] Ridnik T. Ben-Baruch E. Zamir N. Noy A. Friedman I. Protter M. . (2021). “Asymmetric loss for multi-label classification,” in Proceedings of the IEEE/CVF International Conference on Computer Vision (Montreal, QC: IEEE), 82–91.

[B16] Singhal K. Tu T. Gottweis J. Sayres R. Wulczyn E. Amin M. . (2025). Toward expert-level medical question answering with large language models. Nat. Med. 31, 943–950. doi: 10.1038/s41591-024-03423-7 39779926 PMC11922739

[B17] Teng F. Liu Y. Li T. Zhang Y. Li S. Zhao Y. (2023). A review on deep neural networks for ICD coding. IEEE Trans. Knowl. Data Eng. 35, 4357–4375. doi: 10.1109/TKDE.2022.3148267

[B18] Vu T. Nguyen D. Q. Nguyen A. (2020). “A label attention model for icd coding from clinical text,” in Proceedings of the Twenty-Ninth International Joint Conference on Artificial Intelligence, IJCAI-20, eds. C. Bessiere (Montreal: International Joint Conferences on Artificial Intelligence Organization), 3335–3341.

[B19] Wang Z. Wang Y. Zhang H. Wang W. Qi J. Chen J. . (2024). Icdxml: enhancing icd coding with probabilistic label trees and dynamic semantic representations. Scient. Reports 14:18319. doi: 10.1038/s41598-024-69214-9 39112791 PMC11306547

[B20] Wu Y. Zhang J. Chen X. Yao X. Chen Z. (2025). Contrastive learning with large language models for medical code prediction. Expert Syst. Appl. 277:127241. doi: 10.1016/j.eswa.2025.127241

[B21] Yang Y. Lin H. Yang Z. Zhang Y. Zhao D. Luo L. (2025). LCDL: Classification of icd codes based on disease label co-occurrence dependency and longformer with medical knowledge. Artif. Intellig. Med. 160:103041. doi: 10.1016/j.artmed.2024.103041 39667117

[B22] Yang Z. Wang S. Rawat B. P. S. Mitra A. Yu H. (2022). “Knowledge injected prompt based fine-tuning for multi-label few-shot ICD coding,” in Findings of the Association for Computational Linguistics: EMNLP 2022, eds. Y. Goldberg, Z. Kozareva, and Y. Zhang (Abu Dhabi: Association for Computational Linguistics). 36848298

[B23] Yuan Z. Tan C. Huang S. (2022). “Code synonyms do matter: Multiple synonyms matching network for automatic ICD coding,” in Proceedings of the 60th Annual Meeting of the Association for Computational Linguistics, eds. S. Muresan, P. Nakov, and A. Villavicencio (Dublin: Association for Computational Linguistics).

[B24] Zhang J. Li C. Kosov S. Grzegorzek M. Shirahama K. Jiang T. . (2021). Lcu-net: A novel low-cost U-Net for environmental microorganism image segmentation. Pattern Recognit. 115:107885. doi: 10.1016/j.patcog.2021.107885

[B25] Zhang J. Li C. Yin Y. Zhang J. Grzegorzek M. (2023). Applications of artificial neural networks in microorganism image analysis: a comprehensive review from conventional multilayer perceptron to popular convolutional neural network and potential visual transformer. Artif. Intell. Rev. 56:1013–1070. doi: 10.1007/s10462-022-10192-7 35528112 PMC9066147

[B26] Zhang J. Liu L. Gao K. Hu D. (2025a). A forward and backward compatible framework for few-shot class-incremental pill recognition. IEEE Trans. Neural Networks Learn. Syst. 36, 9837–9851. doi: 10.1109/TNNLS.2024.3497956 40030571

[B27] Zhang J. Liu L. Silvén O. Pietikäinen M. Hu D. (2025b). Few-shot class-incremental learning for classification and object detection: a survey. IEEE Trans. Pattern Anal. Mach. Intell. 47, 2924–2945. doi: 10.1109/TPAMI.2025.3529038 40031007

[B28] Zhang J. Zhao P. Zhao Y. Li C. Hu D. (2025c). Few-shot class-incremental learning for retinal disease recognition. IEEE J. Biomed. Health Inform. 29, 9001–9012. doi: 10.1109/JBHI.2024.3457915 39292587

